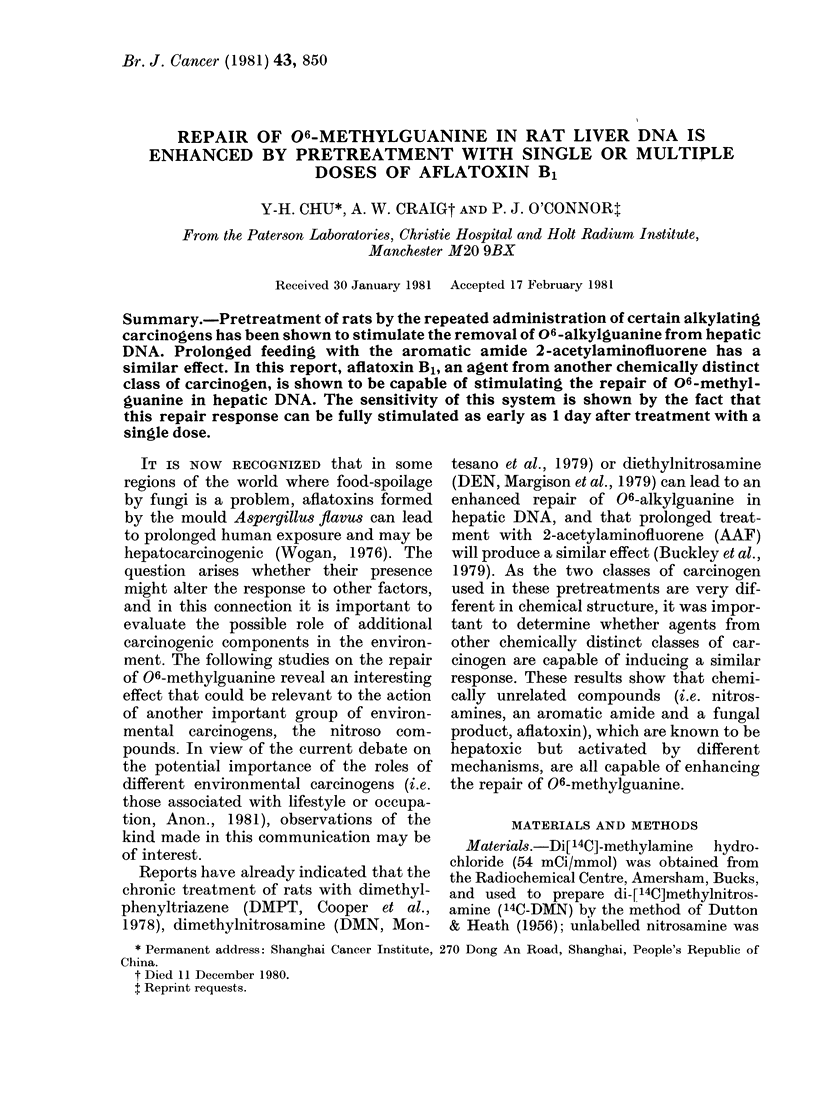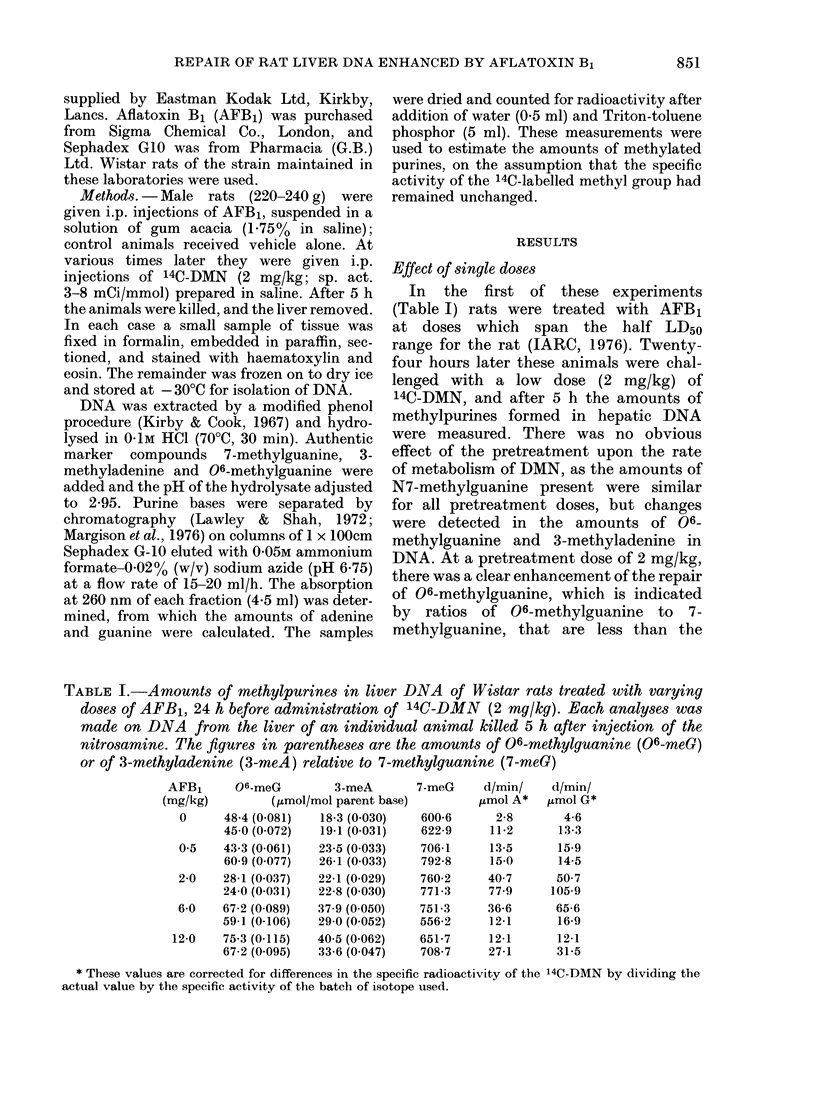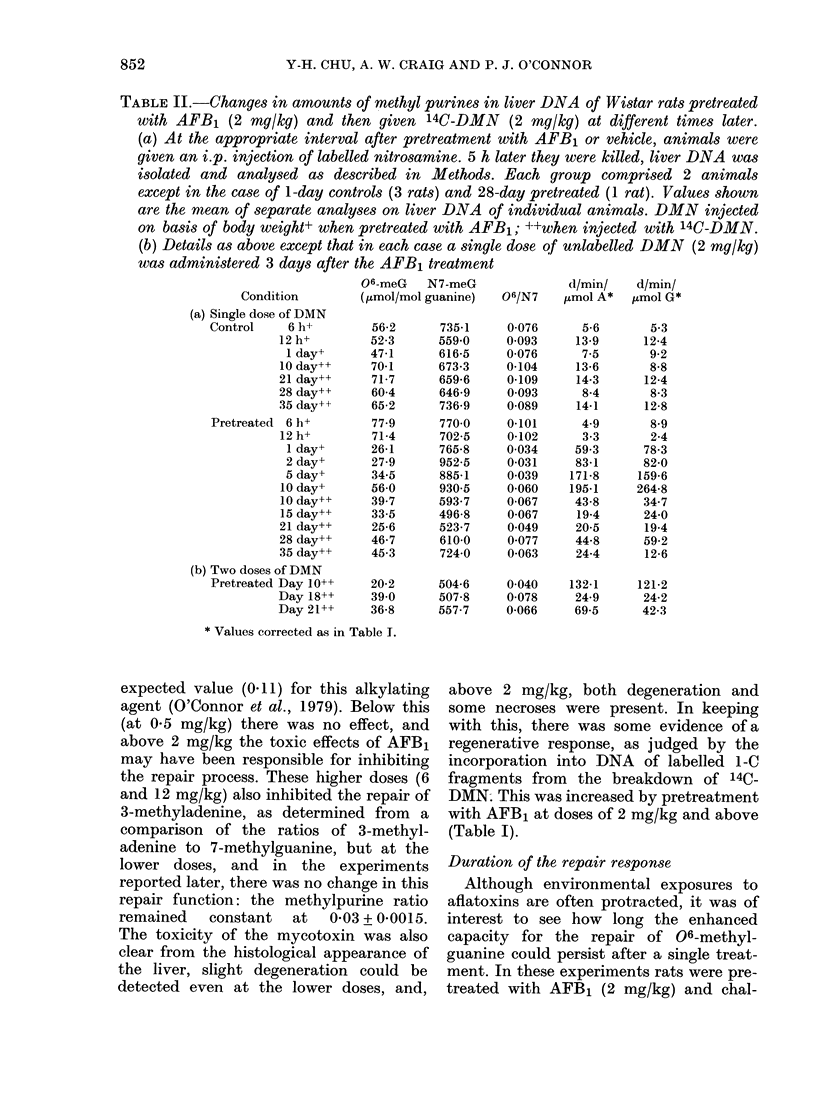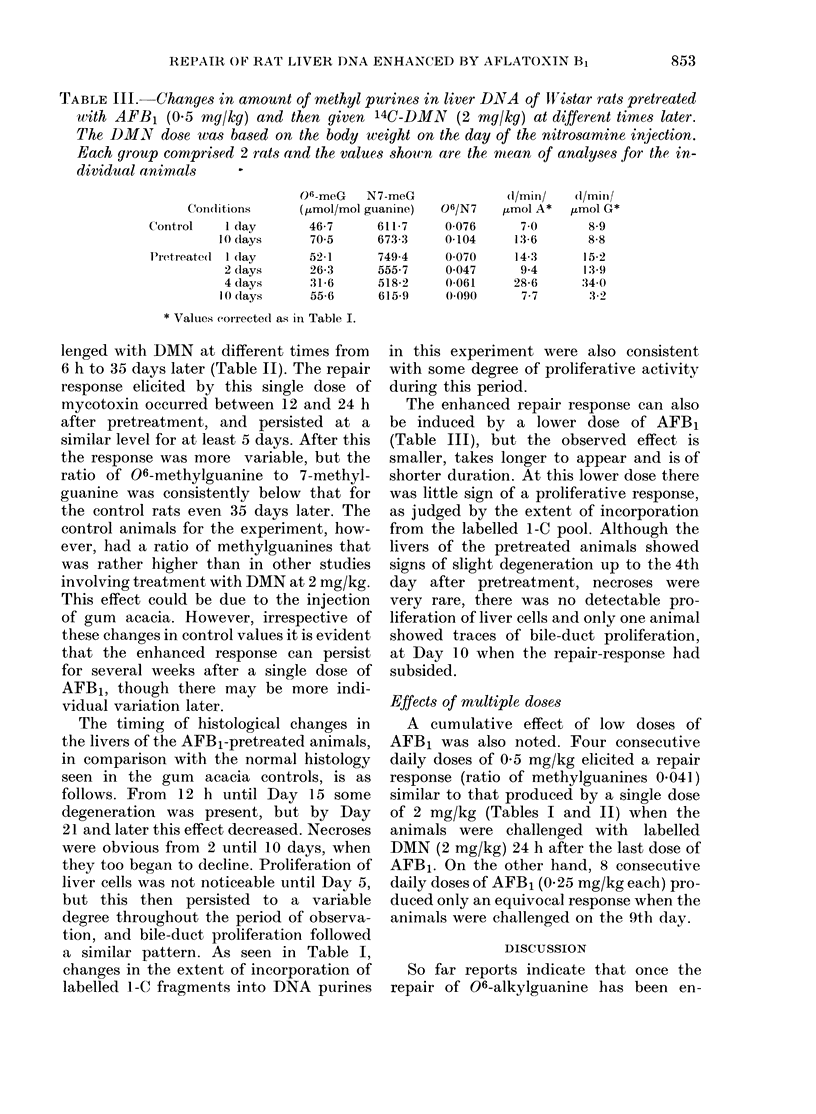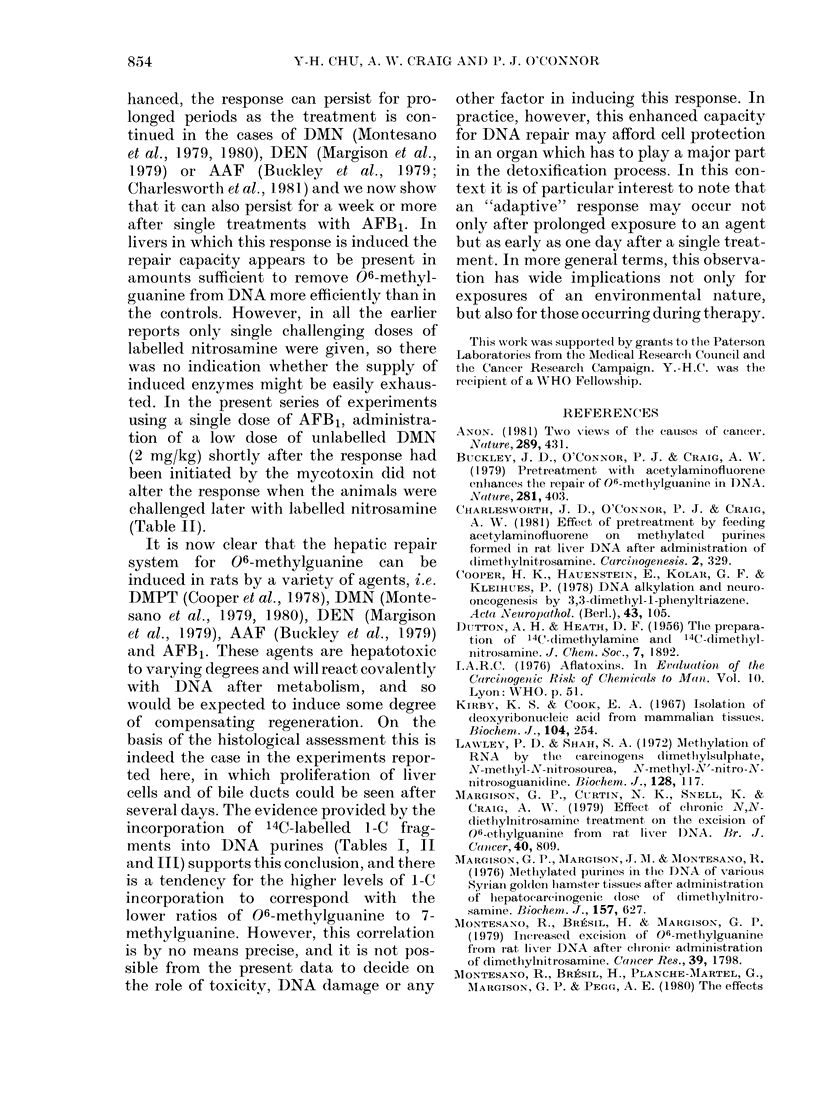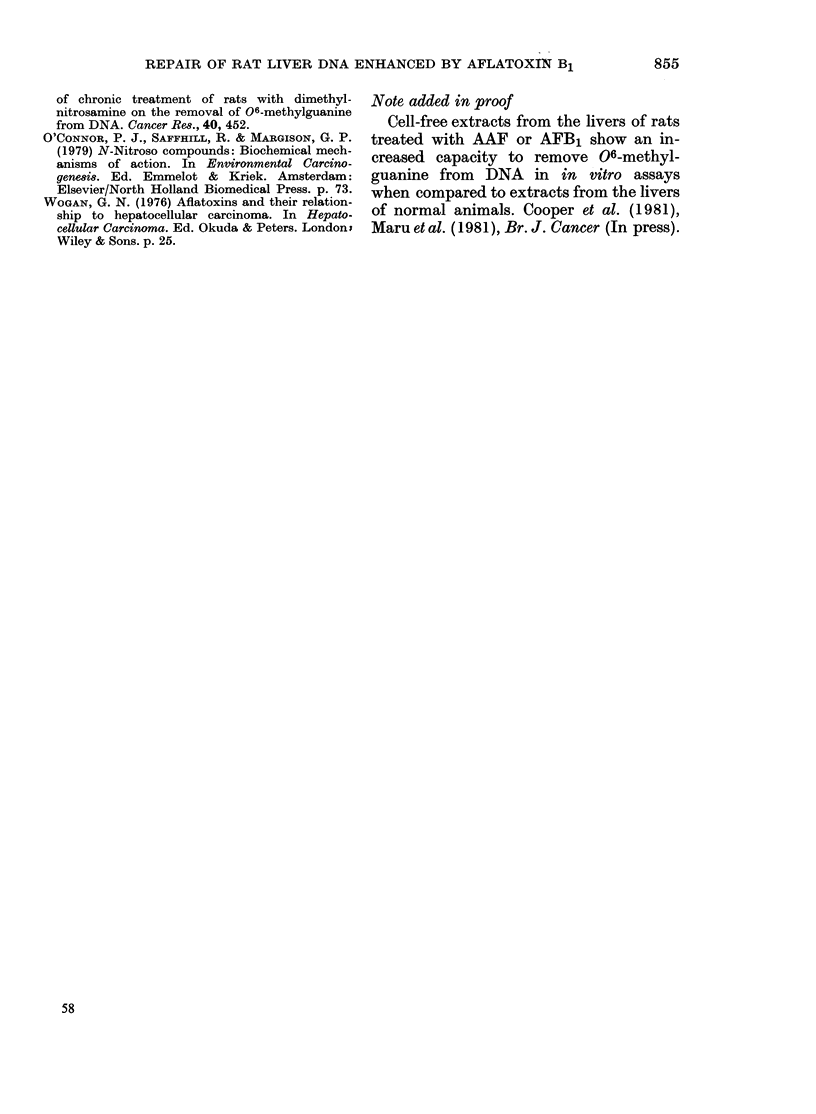# Repair of O6-methylguanine in rat liver DNA is enhanced by pretreatment with single or multiple doses of aflatoxin B1.

**DOI:** 10.1038/bjc.1981.124

**Published:** 1981-06

**Authors:** Y. H. Chu, A. W. Craig, P. J. O'Connor

## Abstract

Pretreatment of rats by the repeated administration of certain alkylating carcinogens has been shown to stimulate the removal of O6-alkylguanine from hepatic DNA. Prolonged feeding with the aromatic amide 2-acetylaminofluorene has a similar effect. In this report, aflatoxin B1, an agent from another chemically distinct class of carcinogen, is shown to be capable of stimulating the repair of O6-methylguanine in hepatic DNA. The sensitivity of this system is shown by the fact that this repair response can be fully stimulated as early as 1 day after treatment with a single dose.


*Br. J. Cancer* (1981) **43**, 850

### REPAIR OF $O^6$-METHYLGUANINE IN RAT LIVER DNA IS ENHANCED BY PRETREATMENT WITH SINGLE OR MULTIPLE DOSES OF AFLATOXIN $B_1$

Y-H. CHU*, A. W. CRAIG† AND P. J. O'CONNOR‡

*From the Paterson Laboratories, Christie Hospital and Holt Radium Institute, Manchester M20 9BX*

Received 30 January 1981 Accepted 17 February 1981

**Summary.—Pretreatment of rats by the repeated administration of certain alkylating carcinogens has been shown to stimulate the removal of $O^6$-alkylguanine from hepatic DNA. Prolonged feeding with the aromatic amide 2-acetylaminofluorene has a similar effect. In this report, aflatoxin $B_1$, an agent from another chemically distinct class of carcinogen, is shown to be capable of stimulating the repair of $O^6$-methylguanine in hepatic DNA. The sensitivity of this system is shown by the fact that this repair response can be fully stimulated as early as 1 day after treatment with a single dose.**

IT IS NOW RECOGNIZED that in some regions of the world where food-spoilage by fungi is a problem, aflatoxins formed by the mould *Aspergillus flavus* can lead to prolonged human exposure and may be hepatocarcinogenic (Wogan, 1976). The question arises whether their presence might alter the response to other factors, and in this connection it is important to evaluate the possible role of additional carcinogenic components in the environment. The following studies on the repair of $O^6$-methylguanine reveal an interesting effect that could be relevant to the action of another important group of environmental carcinogens, the nitroso compounds. In view of the current debate on the potential importance of the roles of different environmental carcinogens (*i.e.* those associated with lifestyle or occupation, Anon., 1981), observations of the kind made in this communication may be of interest.

Reports have already indicated that the chronic treatment of rats with dimethylphenyltriazene (DMPT, Cooper *et al.*, 1978), dimethylnitrosamine (DMN, Montesano *et al.*, 1979) or diethylnitrosamine (DEN, Margison *et al.*, 1979) can lead to an enhanced repair of $O^6$-alkylguanine in hepatic DNA, and that prolonged treatment with 2-acetylaminofluorene (AAF) will produce a similar effect (Buckley *et al.*, 1979). As the two classes of carcinogen used in these pretreatments are very different in chemical structure, it was important to determine whether agents from other chemically distinct classes of carcinogen are capable of inducing a similar response. These results show that chemically unrelated compounds (*i.e.* nitrosamines, an aromatic amide and a fungal product, aflatoxin), which are known to be hepatoxic but activated by different mechanisms, are all capable of enhancing the repair of $O^6$-methylguanine.

### MATERIALS AND METHODS

*Materials.*—Di[$^{14}$C]-methylamine hydrochloride (54 mCi/mmol) was obtained from the Radiochemical Centre, Amersham, Bucks, and used to prepare di-[$^{14}$C]methylnitrosamine ($^{14}$C-DMN) by the method of Dutton & Heath (1956); unlabelled nitrosamine was

* Permanent address: Shanghai Cancer Institute, 270 Dong An Road, Shanghai, People's Republic of China.

† Died 11 December 1980.

‡ Reprint requests.

supplied by Eastman Kodak Ltd, Kirkby, Lancs. Aflatoxin $B_1$ ($AFB_1$) was purchased from Sigma Chemical Co., London, and Sephadex G10 was from Pharmacia (G.B.) Ltd. Wistar rats of the strain maintained in these laboratories were used.

*Methods.*—Male rats (220–240 g) were given i.p. injections of $AFB_1$, suspended in a solution of gum acacia (1·75% in saline); control animals received vehicle alone. At various times later they were given i.p. injections of $^{14}$C-DMN (2 mg/kg; sp. act. 3–8 mCi/mmol) prepared in saline. After 5 h the animals were killed, and the liver removed. In each case a small sample of tissue was fixed in formalin, embedded in paraffin, sectioned, and stained with haematoxylin and eosin. The remainder was frozen on to dry ice and stored at −30°C for isolation of DNA.

DNA was extracted by a modified phenol procedure (Kirby & Cook, 1967) and hydrolysed in 0·1M HCl (70°C, 30 min). Authentic marker compounds 7-methylguanine, 3-methyladenine and $O^6$-methylguanine were added and the pH of the hydrolysate adjusted to 2·95. Purine bases were separated by chromatography (Lawley & Shah, 1972; Margison *et al.*, 1976) on columns of 1 × 100cm Sephadex G-10 eluted with 0·05M ammonium formate–0·02% (w/v) sodium azide (pH 6·75) at a flow rate of 15–20 ml/h. The absorption at 260 nm of each fraction (4·5 ml) was determined, from which the amounts of adenine and guanine were calculated. The samples were dried and counted for radioactivity after addition of water (0·5 ml) and Triton-toluene phosphor (5 ml). These measurements were used to estimate the amounts of methylated purines, on the assumption that the specific activity of the $^{14}$C-labelled methyl group had remained unchanged.

### RESULTS

### *Effect of single doses*

In the first of these experiments (Table I) rats were treated with $AFB_1$ at doses which span the half $LD_{50}$ range for the rat (IARC, 1976). Twenty-four hours later these animals were challenged with a low dose (2 mg/kg) of $^{14}$C-DMN, and after 5 h the amounts of methylpurines formed in hepatic DNA were measured. There was no obvious effect of the pretreatment upon the rate of metabolism of DMN, as the amounts of N7-methylguanine present were similar for all pretreatment doses, but changes were detected in the amounts of $O^6$-methylguanine and 3-methyladenine in DNA. At a pretreatment dose of 2 mg/kg, there was a clear enhancement of the repair of $O^6$-methylguanine, which is indicated by ratios of $O^6$-methylguanine to 7-methylguanine, that are less than the

TABLE I.—*Amounts of methylpurines in liver DNA of Wistar rats treated with varying doses of $AFB_1$, 24 h before administration of $^{14}$C-DMN (2 mg/kg). Each analyses was made on DNA from the liver of an individual animal killed 5 h after injection of the nitrosamine. The figures in parentheses are the amounts of $O^6$-methylguanine ($O^6$-meG) or of 3-methyladenine (3-meA) relative to 7-methylguanine (7-meG)*

| $AFB_1$ (mg/kg) | $O^6$-meG | 3-meA | 7-meG | d/min/ μmol A* | d/min/ μmol G* |
|---|---|---|---|---|---|
| | (μmol/mol parent base) | | | | |
| 0 | 48·4 (0·081) | 18·3 (0·030) | 600·6 | 2·8 | 4·6 |
| | 45·0 (0·072) | 19·1 (0·031) | 622·9 | 11·2 | 13·3 |
| 0·5 | 43·3 (0·061) | 23·5 (0·033) | 706·1 | 13·5 | 15·9 |
| | 60·9 (0·077) | 26·1 (0·033) | 792·8 | 15·0 | 14·5 |
| 2·0 | 28·1 (0·037) | 22·1 (0·029) | 760·2 | 40·7 | 50·7 |
| | 24·0 (0·031) | 22·8 (0·030) | 771·3 | 77·9 | 105·9 |
| 6·0 | 67·2 (0·089) | 37·9 (0·050) | 751·3 | 36·6 | 65·6 |
| | 59·1 (0·106) | 29·0 (0·052) | 556·2 | 12·1 | 16·9 |
| 12·0 | 75·3 (0·115) | 40·5 (0·062) | 651·7 | 12·1 | 12·1 |
| | 67·2 (0·095) | 33·6 (0·047) | 708·7 | 27·1 | 31·5 |

* These values are corrected for differences in the specific radioactivity of the $^{14}$C-DMN by dividing the actual value by the specific activity of the batch of isotope used.

TABLE II.—*Changes in amounts of methyl purines in liver DNA of Wistar rats pretreated with $AFB_1$ (2 mg/kg) and then given $^{14}C$-DMN (2 mg/kg) at different times later. (a) At the appropriate interval after pretreatment with $AFB_1$ or vehicle, animals were given an i.p. injection of labelled nitrosamine. 5 h later they were killed, liver DNA was isolated and analysed as described in Methods. Each group comprised 2 animals except in the case of 1-day controls (3 rats) and 28-day pretreated (1 rat). Values shown are the mean of separate analyses on liver DNA of individual animals. DMN injected on basis of body weight$^{+}$ when pretreated with $AFB_1$; $^{++}$when injected with $^{14}C$-DMN. (b) Details as above except that in each case a single dose of unlabelled DMN (2 mg/kg) was administered 3 days after the $AFB_1$ treatment*

| Condition | | $O^6$-meG (μmol/mol guanine) | N7-meG (μmol/mol guanine) | $O^6$/N7 | d/min/ μmol A* | d/min/ μmol G* |
|---|---|---|---|---|---|---|
| (a) Single dose of DMN | | | | | | |
| Control | 6 h$^{+}$ | 56·2 | 735·1 | 0·076 | 5·6 | 5·3 |
| | 12 h$^{+}$ | 52·3 | 559·0 | 0·093 | 13·9 | 12·4 |
| | 1 day$^{+}$ | 47·1 | 616·5 | 0·076 | 7·5 | 9·2 |
| | 10 day$^{++}$ | 70·1 | 673·3 | 0·104 | 13·6 | 8·8 |
| | 21 day$^{++}$ | 71·7 | 659·6 | 0·109 | 14·3 | 12·4 |
| | 28 day$^{++}$ | 60·4 | 646·9 | 0·093 | 8·4 | 8·3 |
| | 35 day$^{++}$ | 65·2 | 736·9 | 0·089 | 14·1 | 12·8 |
| Pretreated | 6 h$^{+}$ | 77·9 | 770·0 | 0·101 | 4·9 | 8·9 |
| | 12 h$^{+}$ | 71·4 | 702·5 | 0·102 | 3·3 | 2·4 |
| | 1 day$^{+}$ | 26·1 | 765·8 | 0·034 | 59·3 | 78·3 |
| | 2 day$^{+}$ | 27·9 | 952·5 | 0·031 | 83·1 | 82·0 |
| | 5 day$^{+}$ | 34·5 | 885·1 | 0·039 | 171·8 | 159·6 |
| | 10 day$^{+}$ | 56·0 | 930·5 | 0·060 | 195·1 | 264·8 |
| | 10 day$^{++}$ | 39·7 | 593·7 | 0·067 | 43·8 | 34·7 |
| | 15 day$^{++}$ | 33·5 | 496·8 | 0·067 | 19·4 | 24·0 |
| | 21 day$^{++}$ | 25·6 | 523·7 | 0·049 | 20·5 | 19·4 |
| | 28 day$^{++}$ | 46·7 | 610·0 | 0·077 | 44·8 | 59·2 |
| | 35 day$^{++}$ | 45·3 | 724·0 | 0·063 | 24·4 | 12·6 |
| (b) Two doses of DMN | | | | | | |
| Pretreated | Day 10$^{++}$ | 20·2 | 504·6 | 0·040 | 132·1 | 121·2 |
| | Day 18$^{++}$ | 39·0 | 507·8 | 0·078 | 24·9 | 24·2 |
| | Day 21$^{++}$ | 36·8 | 557·7 | 0·066 | 69·5 | 42·3 |

* Values corrected as in Table I.

expected value (0·11) for this alkylating agent (O'Connor *et al.*, 1979). Below this (at 0·5 mg/kg) there was no effect, and above 2 mg/kg the toxic effects of $AFB_1$ may have been responsible for inhibiting the repair process. These higher doses (6 and 12 mg/kg) also inhibited the repair of 3-methyladenine, as determined from a comparison of the ratios of 3-methyladenine to 7-methylguanine, but at the lower doses, and in the experiments reported later, there was no change in this repair function: the methylpurine ratio remained constant at $0·03 \pm 0·0015$. The toxicity of the mycotoxin was also clear from the histological appearance of the liver, slight degeneration could be detected even at the lower doses, and, above 2 mg/kg, both degeneration and some necroses were present. In keeping with this, there was some evidence of a regenerative response, as judged by the incorporation into DNA of labelled 1-C fragments from the breakdown of $^{14}C$-DMN. This was increased by pretreatment with $AFB_1$ at doses of 2 mg/kg and above (Table I).

### *Duration of the repair response*

Although environmental exposures to aflatoxins are often protracted, it was of interest to see how long the enhanced capacity for the repair of $O^6$-methylguanine could persist after a single treatment. In these experiments rats were pretreated with $AFB_1$ (2 mg/kg) and chal-

TABLE III.—*Changes in amount of methyl purines in liver DNA of Wistar rats pretreated with $AFB_1$ (0·5 mg/kg) and then given $^{14}C$-DMN (2 mg/kg) at different times later. The DMN dose was based on the body weight on the day of the nitrosamine injection. Each group comprised 2 rats and the values shown are the mean of analyses for the individual animals*

| Conditions | | $O^6$-meG (μmol/mol | N7-meG guanine) | $O^6$/N7 | d/min/ μmol A* | d/min/ μmol G* |
|---|---|---|---|---|---|---|
| Control | 1 day | 46·7 | 611·7 | 0·076 | 7·0 | 8·9 |
| | 10 days | 70·5 | 673·3 | 0·104 | 13·6 | 8·8 |
| Pretreated | 1 day | 52·1 | 749·4 | 0·070 | 14·3 | 15·2 |
| | 2 days | 26·3 | 555·7 | 0·047 | 9·4 | 13·9 |
| | 4 days | 31·6 | 518·2 | 0·061 | 28·6 | 34·0 |
| | 10 days | 55·6 | 615·9 | 0·090 | 7·7 | 3·2 |

* Values corrected as in Table I.

lenged with DMN at different times from 6 h to 35 days later (Table II). The repair response elicited by this single dose of mycotoxin occurred between 12 and 24 h after pretreatment, and persisted at a similar level for at least 5 days. After this the response was more variable, but the ratio of $O^6$-methylguanine to 7-methylguanine was consistently below that for the control rats even 35 days later. The control animals for the experiment, however, had a ratio of methylguanines that was rather higher than in other studies involving treatment with DMN at 2 mg/kg. This effect could be due to the injection of gum acacia. However, irrespective of these changes in control values it is evident that the enhanced response can persist for several weeks after a single dose of $AFB_1$, though there may be more individual variation later.

The timing of histological changes in the livers of the $AFB_1$-pretreated animals, in comparison with the normal histology seen in the gum acacia controls, is as follows. From 12 h until Day 15 some degeneration was present, but by Day 21 and later this effect decreased. Necroses were obvious from 2 until 10 days, when they too began to decline. Proliferation of liver cells was not noticeable until Day 5, but this then persisted to a variable degree throughout the period of observation, and bile-duct proliferation followed a similar pattern. As seen in Table I, changes in the extent of incorporation of labelled 1-C fragments into DNA purines in this experiment were also consistent with some degree of proliferative activity during this period.

The enhanced repair response can also be induced by a lower dose of $AFB_1$ (Table III), but the observed effect is smaller, takes longer to appear and is of shorter duration. At this lower dose there was little sign of a proliferative response, as judged by the extent of incorporation from the labelled 1-C pool. Although the livers of the pretreated animals showed signs of slight degeneration up to the 4th day after pretreatment, necroses were very rare, there was no detectable proliferation of liver cells and only one animal showed traces of bile-duct proliferation, at Day 10 when the repair-response had subsided.

### *Effects of multiple doses*

A cumulative effect of low doses of $AFB_1$ was also noted. Four consecutive daily doses of 0·5 mg/kg elicited a repair response (ratio of methylguanines 0·041) similar to that produced by a single dose of 2 mg/kg (Tables I and II) when the animals were challenged with labelled DMN (2 mg/kg) 24 h after the last dose of $AFB_1$. On the other hand, 8 consecutive daily doses of $AFB_1$ (0·25 mg/kg each) produced only an equivocal response when the animals were challenged on the 9th day.

### DISCUSSION

So far reports indicate that once the repair of $O^6$-alkylguanine has been enhanced, the response can persist for prolonged periods as the treatment is continued in the cases of DMN (Montesano *et al.*, 1979, 1980), DEN (Margison *et al.*, 1979) or AAF (Buckley *et al.*, 1979; Charlesworth *et al.*, 1981) and we now show that it can also persist for a week or more after single treatments with $AFB_1$. In livers in which this response is induced the repair capacity appears to be present in amounts sufficient to remove $O^6$-methylguanine from DNA more efficiently than in the controls. However, in all the earlier reports only single challenging doses of labelled nitrosamine were given, so there was no indication whether the supply of induced enzymes might be easily exhausted. In the present series of experiments using a single dose of $AFB_1$, administration of a low dose of unlabelled DMN (2 mg/kg) shortly after the response had been initiated by the mycotoxin did not alter the response when the animals were challenged later with labelled nitrosamine (Table II).

It is now clear that the hepatic repair system for $O^6$-methylguanine can be induced in rats by a variety of agents, *i.e.* DMPT (Cooper *et al.*, 1978), DMN (Montesano *et al.*, 1979, 1980), DEN (Margison *et al.*, 1979), AAF (Buckley *et al.*, 1979) and $AFB_1$. These agents are hepatotoxic to varying degrees and will react covalently with DNA after metabolism, and so would be expected to induce some degree of compensating regeneration. On the basis of the histological assessment this is indeed the case in the experiments reported here, in which proliferation of liver cells and of bile ducts could be seen after several days. The evidence provided by the incorporation of $^{14}C$-labelled 1-C fragments into DNA purines (Tables I, II and III) supports this conclusion, and there is a tendency for the higher levels of 1-C incorporation to correspond with the lower ratios of $O^6$-methylguanine to 7-methylguanine. However, this correlation is by no means precise, and it is not possible from the present data to decide on the role of toxicity, DNA damage or any other factor in inducing this response. In practice, however, this enhanced capacity for DNA repair may afford cell protection in an organ which has to play a major part in the detoxification process. In this context it is of particular interest to note that an "adaptive" response may occur not only after prolonged exposure to an agent but as early as one day after a single treatment. In more general terms, this observation has wide implications not only for exposures of an environmental nature, but also for those occurring during therapy.

This work was supported by grants to the Paterson Laboratories from the Medical Research Council and the Cancer Research Campaign. Y.-H.C. was the recipient of a WHO Fellowship.

### REFERENCES

ANON. (1981) Two views of the causes of cancer. *Nature*, **289**, 431.

BUCKLEY, J. D., O'CONNOR, P. J. & CRAIG, A. W. (1979) Pretreatment with acetylaminofluorene enhances the repair of $O^6$-methylguanine in DNA. *Nature*, **281**, 403.

CHARLESWORTH, J. D., O'CONNOR, P. J. & CRAIG, A. W. (1981) Effect of pretreatment by feeding acetylaminofluorene on methylated purines formed in rat liver DNA after administration of dimethylnitrosamine. *Carcinogenesis*. **2**, 329.

COOPER, H. K., HAUENSTEIN, E., KOLAR, G. F. & KLEIHUES, P. (1978) DNA alkylation and neurooncogenesis by 3,3-dimethyl-1-phenyltriazene. *Acta Neuropathol.* (Berl.), **43**, 105.

DUTTON, A. H. & HEATH, D. F. (1956) The preparation of $^{14}C$-dimethylamine and $^{14}C$-dimethylnitrosamine. *J. Chem. Soc.*, **7**, 1892.

I.A.R.C. (1976) Aflatoxins. In *Evaluation of the Carcinogenic Risk of Chemicals to Man.* Vol. 10. Lyon: WHO. p. 51.

KIRBY, K. S. & COOK, E. A. (1967) Isolation of deoxyribonucleic acid from mammalian tissues. *Biochem. J.*, **104**, 254.

LAWLEY, P. D. & SHAH, S. A. (1972) Methylation of RNA by the carcinogens dimethylsulphate, *N*-methyl-*N*-nitrosourea, *N*-methyl-*N'*-nitro-*N*-nitrosoguanidine. *Biochem. J.*, **128**, 117.

MARGISON, G. P., CURTIN, N. K., SNELL, K. & CRAIG, A. W. (1979) Effect of chronic *N*,*N*-diethylnitrosamine treatment on the excision of $O^6$-ethylguanine from rat liver DNA. *Br. J. Cancer*, **40**, 809.

MARGISON, G. P., MARGISON, J. M. & MONTESANO, R. (1976) Methylated purines in the DNA of various Syrian golden hamster tissues after administration of hepatocarcinogenic dose of dimethylnitrosamine. *Biochem. J.*, **157**, 627.

MONTESANO, R., BRÉSIL, H. & MARGISON, G. P. (1979) Increased excision of $O^6$-methylguanine from rat liver DNA after chronic administration of dimethylnitrosamine. *Cancer Res.*, **39**, 1798.

MONTESANO, R., BRÉSIL, H., PLANCHE-MARTEL, G., MARGISON, G. P. & PEGG, A. E. (1980) The effects of chronic treatment of rats with dimethylnitrosamine on the removal of $O^6$-methylguanine from DNA. *Cancer Res.*, **40**, 452.

O'CONNOR, P. J., SAFFHILL, R. & MARGISON, G. P. (1979) *N*-Nitroso compounds: Biochemical mechanisms of action. In *Environmental Carcinogenesis*. Ed. Emmelot & Kriek. Amsterdam: Elsevier/North Holland Biomedical Press. p. 73.

WOGAN, G. N. (1976) Aflatoxins and their relationship to hepatocellular carcinoma. In *Hepatocellular Carcinoma*. Ed. Okuda & Peters. London: Wiley & Sons. p. 25.

*Note added in proof*

Cell-free extracts from the livers of rats treated with AAF or $AFB_1$ show an increased capacity to remove $O^6$-methylguanine from DNA in *in vitro* assays when compared to extracts from the livers of normal animals. Cooper *et al.* (1981), Maru *et al.* (1981), *Br. J. Cancer* (In press).